# Explanatory and Apologetic

**Published:** 1898-02

**Authors:** 


EXPLANATORY AND APOLOGETIC.—We had prepared and put in type for this issue an announcement of the big quarantine convention at Mobile, Feb. 9th; giving the programme in full; expecting, of course, to place the JOURNAL in the hands of readers in time to give them the information,—our usual date of issue being 1st to 4th of each month. But owing to delays at the printing office, which were unavoidable, the issue of this number was retarded until too late to make the programme and other matter relating to the convention of any interest; the convention was over before we were out. We hope to be able to make amends in our next issue by giving an account of what was done.

Dr. Swearingen, State Health Officer of Texas, was one of the Executive Committee, but was not able to attend the convention nor to take an active part in the organization. Texas was well represented, however. The Texas delegates were: Drs.

H. A. West, of Galveston; Dr. S. W. Scholars, of Orange; Dr. C. M. Hargrave, of Marshall; Mayor Rice of Houston, and— others whose names escaped us at the moment. The occasion was one of great importance, and we look for some good to result, in the way of protective reforms—much needed.

